# Evaluation of Stomatognathic Problems in Children with Osteogenesis Imperfecta (*Osteogenesis Imperfecta* − *OI*) − Preliminary Study

**DOI:** 10.34763/devperiodmed.20172102.144153

**Published:** 2017-08-11

**Authors:** Danuta Smoląg, Małgorzata Kulesa-Mrowiecka, Jerzy Sułko

**Affiliations:** 1 Medical Department, Jagiellonian University Collegium Medicum, Kraków, Poland; 2 Institute of Physiotherapy, Health Science Department, Jagiellonian University, Kraków, Poland; 3 Child Surgery Department, Polish-American Institute of Pediatrics, Jagiellonian University Collegium Medicum, Kraków, Poland

**Keywords:** stomatognathic system, osteogenesis imperfecta (OI), układ stomatognatyczny, wrodzona łamliwość kości (osteogenesis imperfecta OI)

## Abstract

According to epidemiological data, muscular dysfunctions of the masticatory system occur in 15-23% of the population. Preventive examinations of functional disorders of the stomatognathic system are, therefore, of particular importance. A distinct group of patients exposed to dysfunctions in the area of the masticatory organ locomotor apparatus comprises those with genetic diseases characterised by disorders in collagen formation. One of such diseases is osteogenesis imperfecta (OI) and dentinogenesis imperfecta that usually goes together with the former.

**Aim:**

The objective of this work was to evaluate the frequency with which particular disorders of the masticatory organ locomotor apparatus occur within the group of patients with osteogenesis imperfecta.

**Material and methods:**

The study was performed on patients of the Orthopaedic Clinic of the Polish-American Paediatric Institute in Kraków. The mean age of the children was 7.9 years. In all the cases, a genetic diagnosis of OI has been confirmed. The research methods were based on an in-depth interview on family diseases, pregnancy, postnatal period, feeding, subjective assessment of dysfunctions in the stomatognathic system.

An examination of the deformations in the stomatognathic system and the skeleton was conducted, as well as an examination of the trauma and tone of the jaw.

The relationship between breastfeeding and swallowing and speech disorders was also evaluated. The impact of intubation on mandibular ranges was investigated.

**Results:**

The results obtained were subjected to statistical analysis on the basis of which conclusions were drawn concerning disorders in the stomatognathic system which tend to occur in children with OI. The renunciation of breastfeeding significantly contributes to sucking and swallowing disorders, rumen disorders, as well as biomechanical disorders in the temporomandibular joint. A significant dependence between breastfeeding and swallowing problems was found, whereas there was no such dependence with respect to speech impediments.

**Conclusions:**

The results of the research conducted led to the following conclusions: 1. Among pediatric patients with OI there are disorders in the stomatognathic system. The most common dysfunctions are: abdominal, swallowing and sucking disorders, abnormal muscle structure of the rumen and biomechanical disorders in the temporomandibular joints. Breastfeeding significantly contributes to swallowing disorders. 2. The therapeutic process involving children with OI requires the cooperation of specialists in orthopedics, pediatrics, physiotherapy, orthodontics and neurologopedics to carry out comprehensive diagnostics and treatment tailored to the individual needs of the patient. 3. In order to draw final conclusions, there is a need for more research by means of objective tools, such as EMG and a condensate recorder.

## Streszczenie

Dysfunkcje układu ruchowego narządu żucia, według danych epidemiologicznych, dotyczą 15-23% populacji. Szczególne znaczenie mają badania profilaktyczne czynnościowych zaburzeń układu stomatognatycznego. Specyficzną grupę pacjentów narażonych na dysfunkcje w obrębie układu ruchu narządu żucia stanowią pacjenci z chorobami genetycznymi charakteryzującymi się zaburzeniami w budowie kolagenu. Chorobą taką jest między innymi osteogenesis imperfecta (OI) i towarzyszące jej dentinogenesis imperfecta.

**Cel:**

Celem pracy była ocena częstości i rodzaju występowania poszczególnych zaburzeń układu ruchowego narządu żucia w grupie pacjentów z Osteogenesis Imperfecta.

**Materiał i metody:**

Badaniami objęto pacjentów Poradni Ortopedycznej Ruchowego narządu sto Polsko-Amerykańskiego Instytutu Pediatrii (CMUJ) w Krakowie, średni wiek dzieci wynosił 7,9 lat; u wszystkich potwierdzono genetycznie rozpoznanie OI. Metodami badawczymi były: wywiad oparty o autorską ankietę dotyczącą chorób w rodzinie, przebiegu ciąży, okresu postnatalnego, karmienia, subiektywnej oceny dysfunkcji w układzie stomatognatycznym.

Przeprowadzono również badanie przedmiotowe oceniające deformacje w układzie stomatognatycznym oraz szkielecie, a także badanie toru i zakresu ruchów żuchwy. Analizowano także zależności pomiędzy karmieniem piersią a zaburzeniami połykania i mowy. Badano wpływ intubacji na zakresy ruchów żuchwy.

**Wyniki:**

Otrzymane wyniki poddano analizie statystycznej. Wśród pacjentów pediatrycznych z OI występują zaburzenia w układzie stomatognatycznym; najczęstszymi dysfunkcjami są: zaburzenia w zakresie ssania i połykania, nieprawidłowa struktura mięśnia żwacza oraz zaburzenia biomechaniczne w stawach skroniowo-żuchwowych. Stwierdzono istotną zbieżność między karmieniem piersią a zaburzeniami połykania, natomiast nie było takich zależności w stosunku do zaburzeń mowy.

**Wnioski:**

Wyniki przeprowadzonego badania pozwoliły na wyciągnięcie następujących wniosków: 1. Wśród pacjentów pediatrycznych z OI występują zaburzenia w układzie stomatognatycznym. Najczęstszymi dysfunkcjami są: zaburzenia w zakresie ssania i połykania, nieprawidłowa struktura mięśnia żwacza oraz zaburzenia biomechaniczne w stawach skroniowo-żuchwowych. Zaniechanie karmienia piersią istotnie przyczynia się do wystąpienia zaburzeń połykania. 2. Proces leczniczy obejmujący dzieci z OI wymaga współpracy specjalistów z zakresu ortopedii, pediatrii, fizjoterapii, ortodoncji oraz neurologopedii w celu przeprowadzenia kompleksowej diagnostyki oraz prowadzenia terapii dostosowanej do indywidualnych potrzeb pacjenta. 3. Konieczne są dalsze badania przy użyciu obiektywnych narzędzi jakim jest EMG oraz zapis kondylografii co pozwoli wyciągnąć wiążące wnioski.

## Introduction

According to epidemiological data, muscular dysfunctions of the masticatory system occur in 15-23% of the general population. As we know from many clinical studies, the percentage of patients who suffer from dysfunctions in the stomatognathic system is 50-80%. Among persons suffering from symptoms of temporomandibular joint disorders, the female sex predisposes to such disorders in the proportion of 2:1 or even 3:1 [1, 2]. So far studies of functional disorders in the area of the stomatognathic system have mainly been related to the adult population. There are very few publications available concerning children. Some of the most significant ones include works that discuss the impact of the occlusion plane upon body posture, occlusion disorders, or displacement of impacted permanent teeth [18, 19, 20]. Due to low awareness among the society of the occurrence of disorders in the masticatory organ area in children and adolescents, it is recommended that educational health-promotion activities through broadly-comprehended preventive care extending to both adults and children should be undertaken. Preventive examinations of functional disorders of the stomatognathic system are of particular importance [3]. Results of screening studies suggest that the age of the persons who suffer from temporomandibular joint diseases is increasingly lower [4, 5].

It is also significant that temporomandibular joint disorders are not only limited to dysfunctions of the joint itself and its periarticular structures; they also extend to structures located far away from the source of pain. The main reasons for dysfunctions of the masticatory organ locomotor apparatus include inter alia: joint stiffness condition, mandibular injuries, increased or decreased muscle tension, body posture disorders, and afferent impulses connected with the feeling of deep pain.

A distinct group of patients exposed to dysfunction in the area of the masticatory organ locomotor apparatus comprises those with genetic diseases characterised by collagen formation disorders. One of such diseases is osteogenesis imperfecta (OI), as well as dentinogenesis imperfecta and dentin dysplasia, which accompany the former. Patients who suffer from this disease reveal distorted proportions and symmetry of the body, uneven distribution of connective tissue, osteoarticular deformations, ligament laxity, vascular lesions, improper construction of auditory ossicles, neurological disorders, as well as shortened and deformed tooth roots, and thin, brittle tooth enamel. The consequence is a frequent occurrence of: lowering of the occlusion height, occlusion defects, and functional disorders of the masticatory organ.

In works devoted to this disease relatively little attention is given to the occurrence of stomatognathic problems and functional disorders of the masticatory organ, i.e. biting and chewing of foods, swallowing of foods, and joint participation in breathing and speech functions due to the crossing of the digestive tract with the respiratory tract. Definitions related to the stomatognathic system concept are presented in table I.

**Table I:** Definitions of terms by Medical Subject Headings (MeSH). Tabela I. Definicje pojęć wg Medical Subject Headings (MeSH).

Concept	Definition
Stomatognathic system	A morpho-functional whole that consists of dental, osteoarticular, muscular structures and glands, connected with one another by means of blood vessels, lymphatic vessels and nerve fibres of the somatic and vegetative system; the stomatognathic system consists of such elements as: the oral cavity, teeth, the jaw, the mandible, the pharynx, and structures connected with chewing, swallowing and speech functions
Masticatory organ	Masticatory organ locomotor apparatus (MOLA), suprahyoid muscles, the tongue, lips, cheeks, salivary glands and dento-maxillary system (DMS).
Masticatory organ locomotor apparatus	Mandible connected with the cranium in the temporomandibular joint (TMJ), including masticatory muscles (temporal, masseter, medial pterygoid, upper lateral pterygoid, and lower lateral pterygoid).
Dental organ	Teeth including periodontium

## Aim

Assessment of the frequency of particular disorders of the masticatory organ locomotor apparatus within the group of patients with osteogenesis imperfecta, and an attempt at assessment of their impacts upon child development.

## Material and methods

The study included 19 children from Poradnia Ortopedyczna Polsko-Amerykańskiego Instytutu Pediatrii (UJ CM) in Kraków with genetically diagnosed osteogenesis imperfecta. A group of 20 patients with osteogenesis imperfecta was selected (one child was excluded from the study in the process due to psychomotoric disorders that made it impossible to perform the physical examination). The basic condition for a patient to qualify for the study was the diagnosis of osteogenesis imperfecta . Another selection criterion was the patient’s age which had to range from 1 to 18 years. The criterion that excluded the patient from the test group was the coexistence of a serious psychomotoric disability that made it impossible to perform the physical examination. The above-mentioned patient selection criteria were used to restrict the number of any disturbance factors and in this way achieve the best possible homogeneity of the group.

The study included children whose parents expressed their written consent to perform the necessary procedures upon having received complete information on the project. Children’s medical histories were gathered, covering: diseases that occurred in their families, the course of pregnancy, perinatal and breastfeeding disorders.

Our own questionnaire form was also filled in, covering: the course of the disease and the current state of the child’s health, subjective assessment of dysfunctions in the stomatognathic system, and assessment of pain.

**Fig. 1:**
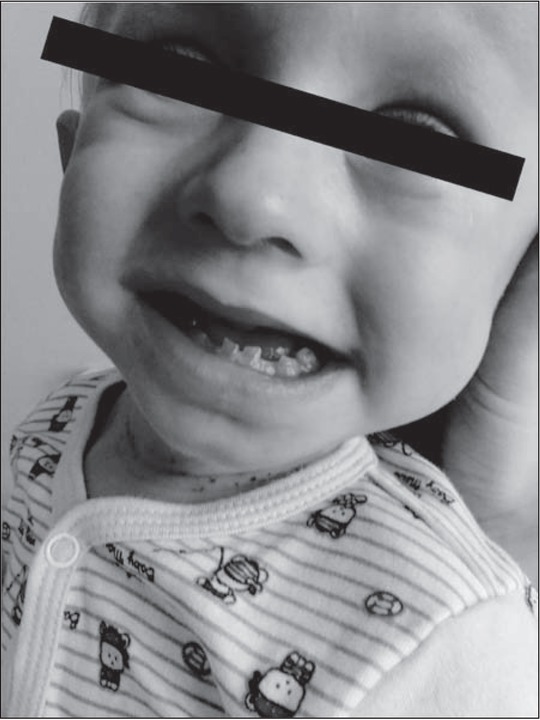
Deviation of the mandible toward the dysfunctional left temporomandibular joint in a 2.5-year old-boy. Ryc. 1. Zbaczanie żuchwy w kierunku dysfunkcyjnego lewego stawu skroniowo-żuchwowego u 2,5-letniego chłopca. (źródło własne)

**Fig. 2:**
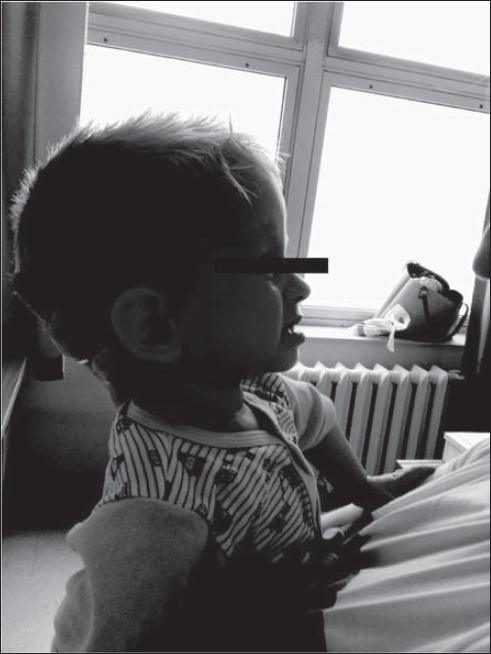
Mandibular protrusion of the emerging cross-bite or undershot in a 2.5-year-old boy. Ryc. 2. Protruzja żuchwy z kształtującym się zgryzem krzyżowym i przodozgryzem u chłopca 2,5 roku. (źródło własne)

**Fig. 3:**
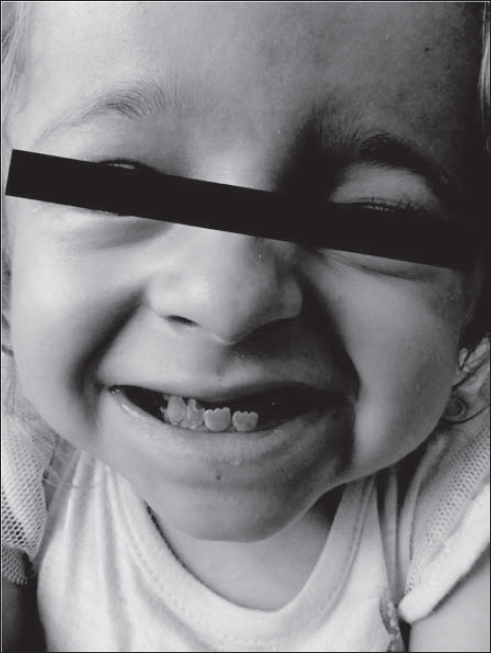
Protrusion of the jaw with cross bite and undershot in a 7-year-old girl with dentin dysplasia. Ryc. 3. Protruzja żuchwy ze zgryzem krzyżowym i przodozgryzem u 7-letniej dziewczynki z dysplazją zębiny. (źródło własne)

**Fig. 4:**
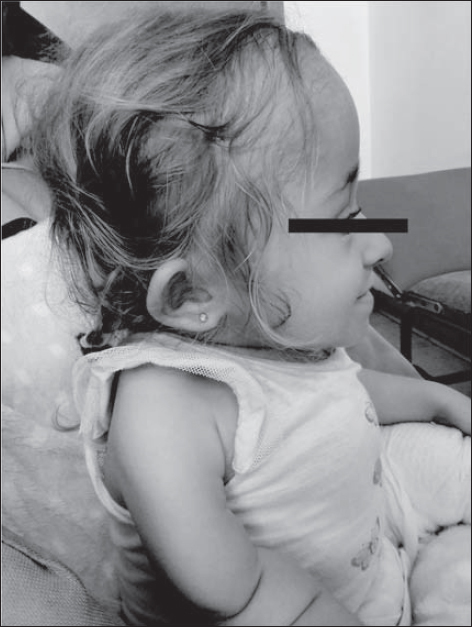
Shortening of the cervical spine. Protrusion of the jaw with cross bite and undershot in a-7-year-old girl with dentin dysplasia. Ryc. 4. Skrócenie odcinka szyjnego kręgosłupa. Protruzja żuchwy ze zgryzem krzyżowym i przodozgryzem u 7-letniej dziewczynki z dysplazją zębiny. (źródło własne)

**Fig. 5:**
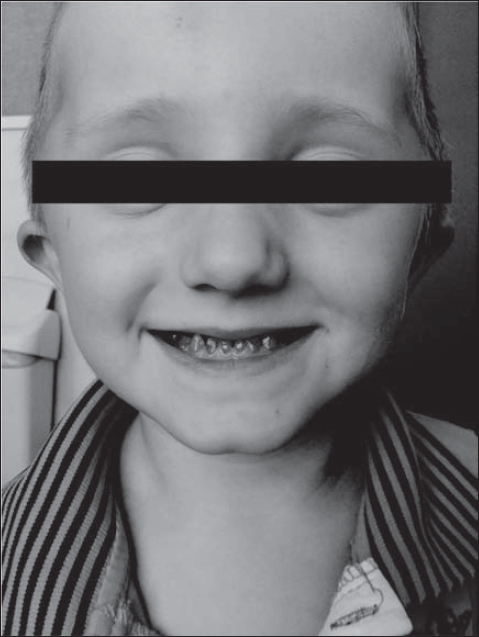
Dentin dysplasia and bone deformity of the skull in a 5-year-old boy with OI. Ryc. 5. Dysplazja zębiny ze zniekształceniem kości czaszki u 5-letniego chłopca z OI. (źródło własne)

Photos in 5 views were taken to illustrate some visible anomalies in the osteoarticular system and structural anomalies in the facial skeleton (Fig. 1-5).

**Graph 1:**
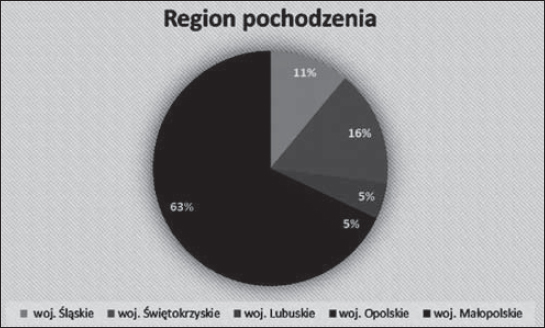
The proportion of respondents by their place of residence Wykres 1. Procentowy podział badanych ze względu na miejsce zamieszkania.

The physical examination comprised:

- postural analysis according to Langlade,
- assessment of bone deformations in the stomatognathic system and skeletal deformations,
- joints, muscles of the masticatory organ, of the neck, and the upper limb girdle,
- examination concerning the occurrence of crepitation, cracking noises,

E. examination of the path and range of mandible movements in all their axes (the maximum mandible retraction range was measured using a rule calibrated with the accuracy of 1 mm.

Both measurements were repeated three times, in several-minute-long time intervals); a front graph was drawn for each patient.

The initial examination of the patient aimed to make an initial assessment of the defects in their body posture and the way they move, including the position of the head and the shoulder girdle. Simultaneously with observation, the above-mentioned medical history was examined. Information upon the course of each pregnancy, the postnatal period, and symptoms of masticatory organ locomotor apparatus dysfunctions and other data which were obtained using the questionnaire and medical history is placed in table II, splitting respondents by their sex and age. The averaged results of mandible movement range measurements, in total and for each age group, are presented in table III.

**Table II:** The occurrence of the characteristic overall and in subgroups of subjects, by gender, tested in percentages. Tabela II. Występowanie badanej cechy u pacjentów ogółem oraz w podgrupach z uwzględnieniem płci, w procentach.

Feature	Total	Girls	Boys
Number of respondents	19 100%	10 52%	9 48%
Average age	7.9	7.4	8.5
Diseases in the patient’s family	47%	50%	44%
Average duration of pregnancy	37	37.3	36.8
Complications %	31%	30%	33%
Natural childbirth	48%	60%	34%
Childbirth cc	52%	40%	66%
APGAR average	8,4	9	8
Breastfeeding	73%	90%	55%
Swallowing disorders	26%	20%	33%
Sucking disorders	42%	40%	44%
Speech disorders	15%	20%	11%
Breathing disorders	10%	0%	22%
Clenching the jaw	15%	20%	11%
Mandibular pain	10%	10%	11%
Headache	5%	10%	0%
Aggravation	5%	10%	0%
Grinding the teeth	15%	10%	22%
Mouth opening problems	15%	20%	11%
Mouth closing problems	10%	0%	22%
Sensitive teeth	15%	20%	11%
Head injuries	5%	0%	11%
Girdle fracture	57%	50%	66%
Intubation	47 %	50%	55%
Aches	10%	0%	22%
Lowered tension	10%	10%	11%
Mandibular path proper	16%	20%	11%
Mandibular path deviated to the right	61%	55%	66%
Mandibular path deviated to the left	23%	25%	23%
Masseter muscle normal /distorted	55%/45%	33%/67%	44%/56%
Crepitation	47%	30%	66%
Bone deformations	21%	10%	33%
Do not move on their own	31%	20%	44%
Proper posture	47%	50%	66%

**Table III:** Averaged results of the measurement ranges of mandibular movements in general and in specific age groups. Tabela III. Uśrednione wyniki pomiarów zakresów ruchów żuchwy ogółem oraz w poszczególnych grupach wiekowych.

Examined feature	Mean movement range in the entire group of respondents	Mean movement range in the girls’ group	Mean movement range in the boys’ group
Retraction	44mm	43mm	42mm
Retraction in the 3-6 year-old age group	45mm		
7-10 years	40mm		
11-15 years	55mm		
16-18 years	40mm		
Protrusion	6 mm	4mm	8mm
Translation to the left	9mm	8mm	10mm
3-6 years	7mm		
7-10	9mm		
11-15	11mm		
16-18	10mm		
Translation to the right	8mm	9mm	8mm
3-6	8mm		
7-10	6mm		
11-15	8mm		
16-18	10mm		

The patient took a free sitting position with the head turned at the angle of 70° in relation to the base plane, and the patient’s facial symmetry, the function of mimic muscles, and mandible movements while speaking were examined.

Periarticular structures of temporomandibular joints were examined by palpation (outside and inside the oral cavity), as were masticatory suboccipital muscles and pectoral muscles. Examination was performed of: the tension degree of the examined muscles; the increased muscle attachment tenderness, enlargements, and the morphological structure of bellies. Examination of the range of mandible movements in all their axes comprised: retraction and protraction movements, protrusion movements and translation movements. Mandible lowering and lifting movements were assessed using the objectified metric method. The mandible retraction range was examined in more depth by performing two measurements: maximum free retraction, i.e. the distance between incisal edges of the teeth and the occlusion line (upon having marked it at the lower incisors). The mandible retraction range was measured using a ruler that was calibrated with the accuracy of 1 mm.

## Results

The results obtained were subjected to a statistical analysis to determine the relationship between the tested features. Chi square tests for independence, t-test for independent samples, and one-way analysis of variance were used. Dependencies between individual features were analyzed using Statistica. In all the tests carried out, a significance level of 0.05 was adopted.

Information on the course of pregnancy, postnatal period and symptoms of dysfunction of the chewing motion system and other data obtained through interview and questionnaire are presented in table II, taking into account the distribution of the tests by gender.

The data show that most of the children with OI were breastfed. Renunciation of breastfeeding significantly contributes to swallowing disorders. 42% of the subjects had suction disorders and 15% speech disorders. It is also interesting that only half of the study group are children born by caesarean section. In the area of masticatory dysfunction 15% of the children have had teeth grinding and 15% teeth clenching, 5% headaches. The mandibular vein was affected by 85% of the majority of children tested.

The averaged results of the measurements of the mandibular movements in general and in the age groups are presented in table III. The mean range of dehydration in the study group was similar in boys and girls. The range of movements in temporomandibular joints does not differ from functional norms, although there is a tendency for hypermobility in temporomandibular joints, also dehydration, protuberance and translation.

The results obtained were subjected to statistical analysis in order to determine the relationships between the features examined. Inter alia, chi square tests of independence, Student’s t test for independent samples, and single variant analysis were used.

In order to examine breastfeeding effects upon swallowing and speech disorders, the chi square test of independence was used. Based upon the test conducted, a statistically significant relationship (p=0.0028) was identified between breastfeeding and swallowing disorders. Breastfeeding discontinuation significantly contributed to the occurrence of swallowing disorders in children with OI (tab. IV). Based upon the test conducted, no occurrence of statistically significant relationship (p=0.7642) was found between breastfeeding and speech disorders (tab. V).

**Tabela IV:** Breast-feeding and swallowing disorders. Tabela IV. Karmienie piersią a zaburzenia połykania.

Breastfeeding	Numbers being examined		
	Proper swallowing	Distorted swallowing	Total
None	1	4	5
%	20.00%	80.00%	
Up to one year	9	0	9
%	100.00%	0.00%	
More than one year	2	0	2
%	100.00%	0.00%	
Total	12	4	16

**Table V:** Breast-feeding and speech impediments. Tabela V. Karmienie piersią a zaburzenia mowy.

Breastfeeding	Numbers being examined		
	Proper speech	Distorted speech	Total
None	4	1	5
%	80.00%	20.00%	
Up to one year	7	2	9
%	77.78%	22.22%	
More than one year	2	0	2
%	100.00%	0.00%	
Total	13	3	16

Intubation effects upon mandible movement ranges due to frequent surgery treatments in children with OI were also examined. Student’s t test for independent samples was used. Based upon the test conducted, no significant difference was found in retraction, protrusion and lateral movements depending on the intubation type used (tab. VI). Table VII presents the results of the analysis of age variance on the extent of the jaw movement. The joints’ range of movement depends on the age of the child.

**Table VI:** The impact of intubation on the scope of the abduction of the mandible. Tabela VI. Wpływ intubacji na zakres odwodzenia żuchwy

Variant	T-test; Group variant: Intubation Grupa 1: No Grupa 2: Yes				
	Mean No	Mean Yes	t	df	p
Abduction	43.00	47.00	-0.7361	15	0.4730

**Table VII:** Effect of age on the range of motion of the jaw. Tabela VII. Wpływ wieku na zakres ruchomości żuchwy

Variable	Variance analysis							
	SS effect	df effect	MS effect	SS error	df error	MS error	F	p
Abduction	480.58	3	160.19	1463.18	13	112.55	1.4233	0.2808
Protrusion	12.08	3	4.03	229.71	15	15.31	0.2628	0.8511
Shift right	24.35	3	8.12	169.43	14	12.10	0.6707	0.5840
Shift left	41.14	3	13.71	180.86	14	12.92	1.0616	0.3967

## Discussion

To-date the study results point to the fact that functional disorders in the area of the stomatognathic system in particular pertain to the adult population. Meta-analysis of 51 studies performed by De Kanter on the frequency in which these disorders occur con( rmed that functional disorders in the stomatognathic system were felt by 30% of patients, whereas they were identified in 44% of the patients in clinical studies [6].

The Study Health Project conducted in Pomerania, which comprised stomatognathic system examinations, showed that approx. 49% of the respondents had at least one symptom that pointed to temporomandibular joint disorders (25%) or masticatory muscle hypersensitivity (15%) [6, 8].

What occurred most frequently were: irregular mandible movements (28%), acoustic symptoms from temporomandibular joints (25%), and masticatory muscle hypersensitivity (15%) [6, 8].

Only few publications are available on the problem we discuss here. Hence, based upon the results of our own initial examinations, it should be considered that that OI-type diseases pose a serious risk factor for the occurrence of the above-mentioned dysfunctions. Most studies are devoted to masticatory organ locomotor apparatus dysfunctions in healthy children and adolescents (who are free from genetic diseases). In her systematic review of literature, Dąbrowska-Gontarczyk presented the epidemiology of bruxism in children and adolescents in the years 2000-2012. As a result of the analysis of 50 publications on randomised clinical studies, cohort studies and clinical/control studies, it was concluded that the frequency of bruxism occurring in developmental age patients in the general population ranged from 1.5% to 90%. Studies were most frequently conducted on children from 3 to 18 years old. The analysis revealed high discrepancies in the results of studies conducted by different authors, which may be due to the research tools used, i.e. mainly a questionnaire that was filled in by parents and guardians. Clinical evaluation of patients was only conducted in 21 studies. Among symptoms that accompanied bruxism, mainly psychological factors were identified, i.e.: stress, depression, neuroticism. Plentiful publications emphasised the co-occurrence of bruxism with ADHD, autism, and sleeping disorders [10]. The share of genetic factors, i.e. the occurrence of family bruxism, was presented in works by Seraj, Chejfetz and other authors [9, 10, 11, 12, 13, 14], although these scholars did not mention any occurrence of bruxism in genetic diseases such as osteogenesis imperfecta.

Other studies performed by Nekora-Azak (2006) on a group of eighteen-year-olds for a representative population of Istambul revealed pain in the mandibular area (31%), clenching the jaw and grinding the teeth (23%), cracking noises in the temporomandibular joint (23.7%). A higher frequency of signs (45-86%) than symptoms (14-36%) of irregularities in the masticatory organ locomotor apparatus are disclosed by the results of studies conducted earlier by: Solberg et al. (1979), Nilner and Lassing (1981), Agerberg and Inkapool (1990), De Kanter et al. (1993), Nourallah and Johansson (1995) [6].

According to the studies by Chłapowska (2004), Kalinowska and Gołębiewska (2008) performed on Polish children, the age of persons who suffer from the temporomandibular joint condition is decreasing. This is indicated by screening and clinical studies. Studies by Alamoudi (1998) conducted on children aged 3-7 years in Saudi Arabia, revealed disorders in 16.53% of the cases, whereas the most frequently identified symptoms were acoustic symptoms from temporomandibular joints (7.8%), and muscle tenderness (6.77%) [6].

The results obtained from various epidemiological studies concerning the frequency with which signs and symptoms of temporomandibular disorders occur in children and teenagers elaborated by Feteih (2006) unveil high cross-population diversification. The highest rates in the range of 50-80% were identified in Germany, Poland, and Sweden; slightly lower, i.e. 30-50% in the United States, Israel, and Finland; and the lowest, i.e. below 30%, in the Netherlands, Japan, China, Bogota, and Saudi Arabia [8]. Interview surveys conducted in Boston among the parents and guardians of children up to the age of 17 showed that bruxism occurred in 38% of young persons, and functional disorders in 5% [10]. Swedish studies conducted in the years 1983, 1993, and 2003 proved that the occurrence of symptoms from temporomandibular joints was growing together with the respondents’ age. In children aged 3 and 5, symptoms occurred very rarely, whereas more than 50% of children aged 10 and 15 showed one symptom of temporomandibular joint disorder or more [6].

Very few authors have discussed the issue of functional disorders of the masticatory organ in genetic diseases. These are mainly focused on Down syndrome [15, 17]. In the work by Młynarska-Zduniak, it was concluded that as many as 94% of the patients revealed masticatory organ dysfunctions within the group of 284 patients with Down syndrome. In systemic diseases, it is only possible to find descriptions of cases in children, in whom muscular dysfunctions of the masticatory system and morphological lesions occur too [21, 22].

In our own studies, besides swallowing disorders, bruxism was diagnosed in 15% of the cases in children with osteogenesis imperfecta, although we plan to perform studies on a larger group in order to determine the frequency and type of occurrence of other masticatory organ dysfunctions in children with OI.

Stomatognathic system dysfunctions in children constitute a significant therapeutic problem for physiotherapists and neurological speech therapists, as well as ear doctors, dentists, and orthopaedic surgeons. In children with osteogenesis imperfecta, rehabilitation problems are highly complex due to the hazard of sprains and fractures occurring for any minor reason. Besides early physiotherapy and neurological speech therapy intervention, breastfeeding seems to be a significant factor, as, to a large degree, it contributes both to the appropriate development of the masticatory organ muscles and to the structure and biomechanics of the temporomandibular joint itself, as demonstrated in our own studies.

It seems necessary to perform studies on a larger group in order to draw any binding conclusions. More attention should also be paid to adaptive mechanisms and bone tissue plasticity in the aspect of intubation and lack of influence upon the functioning of the stomatognathic system in the studied group. Due to the necessity to improve the quality of life of patients with OI in the stomatognathic aspect, a mouth-and-face improvement programme should be created for this group of patients with special attention being paid to dysphagia.

## Conclusions

Disorders in the stomatognathic system occur among paediatric patients with OI. The most frequently occurring dysfunctions include: sucking and swallowing disorders, improper structure of the masseter muscle, and biomechanical temporomandibular joint disorders (including inappropriate mandibular path, subluxation, and temporomandibular hypermobility of the joint).

The healing process covering children with OI requires collaboration between specialists in orthopaedics, paediatrics, physiotherapy, orthodontics and neurological speech therapy, so that comprehensive diagnostics can be conducted, as well as therapy adjusted to individual patients’ needs.

Further studies are planned using objective tools, such as EMG and condylography records, to be able to draw some more binding conclusions.
